# Safety analysis of Ziagen® (abacavir sulfate) in postmarketing surveillance in Japan†

**DOI:** 10.1002/pds.3589

**Published:** 2014-03-03

**Authors:** Tomoko Kurita, Tomomi Kitaichi, Takako Nagao, Toshiyuki Miura, Yoshifumi Kitazono

**Affiliations:** 1 ViiV Healthcare K. K.Tokyo, 151-8566, Japan; 2 Department of Clinical Medicine, Insitute of Tropical Medicine, Nagasaki UniversityNagasaki, 852-8523, Japan

**Keywords:** abacavir, human immunodeficiency virus, hypersensitivity, myocardial infarction, adverse drug reactions, Japanese, pharmacoepidimiology

## Abstract

**Purpose:**

Abacavir is a nucleoside reverse transcriptase inhibitor indicated for human immunodeficiency virus (HIV) infection. In Japan, Ziagen® (300-mg abacavir sulfate) has been marketed since 1999. To obtain safety data on Ziagen, a mandatory postmarketing surveillance was conducted between September 1999 and September 2009.

**Methods:**

A joint survey [HIV-related Drug Surveys (HRD)] has been conducted involving manufacturers of drugs for HIV treatment in Japan. Safety data from total 643 cases (1345.7 person-years) registered to the HRD surveys and received Ziagen were obtained. Adverse drug reaction (ADR) was defined as adverse event of which association with abacavir could not be “ruled out.”

**Results:**

It was found that the overall frequency of ADR was 47.6% (306/643); the common ADRs were “hyperlipidemia,” “nausea,” “increased γ-glutamyltransferase level,” “increased blood triglycerides,” “abnormal hepatic function,” and so on. Serious adverse events were reported in 65 subjects; however, none of the three fatal cases were clearly associated with Ziagen use. The survey-defined hypersensitivity has been infrequently reported in 15 subjects (2.3%). Although some studies had indicated of the association between abacavir and myocardial infarction, no ischemic heart diseases were reported in the present survey. Two of the three pregnant cases delivered normal neonates (one induced abortion).

**Conclusions:**

During the mandatory postmarketing survey of Ziagen, there were no cases of ischemic heart diseases, and the incidence of hypersensitivity was considerably low. These indicated that abacavir can be safely used in Japanese HIV+ population. However, the safety profile of Ziagen should be continued to be monitored through pharmacovigilance.

## INTRODUCTION

Abacavir sulfate (ABC) is a nucleoside reverse transcriptase inhibitor developed by Glaxo Wellcome (GlaxoSmithKline since 2001), which inhibits replication of human immunodeficiency virus type 1 (HIV-1). Non-clinical studies have shown the following mechanism of action of this drug: the drug is converted to carbovir triphosphate in cells, an active metabolite, and is incorporated into viral deoxyribonucleic acid (DNA), by competing with deoxyguanosine triphosphate, a natural substrate, and it inhibits 5′–3′ phosphodiester bond formation, resulting in inhibition of viral DNA extension.^1^ Many clinical trials have demonstrated the efficacy of combinational anti-retroviral therapy (cART)-containing ABCs.^2^–^12^ Since 2012, abacavir has been listed as an important component of the cART regimens recommended by the international guidelines^13^–^15^ and by the Japanese guideline as well.^16^ In the USA, abacavir was approved for HIV infection on 17 December 1998, and as of February 2010, it has been approved in 88 countries in the world. In Japan, ABC has been marketed with the trade name of “Ziagen 300 mg” since September 1999. During the approval review process in Japan, the drug was privileged priority inspection as a therapeutic drug for HIV infection and promptly reviewed by screening overseas data on the premise that “necessary measures to ensure the safety of patients would be taken after approval” and that “the approved contents of the drug, such as dosage and administration, would be confirmed for use in Japan.” Therefore, a postmarketing surveillance was needed to collect information on the use of this drug to obtain approval. Therefore, between September 1999 and September 2009, we conducted a postmarketing surveillance in 646 patients who received Ziagen tablet at 33 institutions in Japan. Herein, we report the results on safety and effectiveness of this tablet obtained from the postmarketing surveillance. The safety data presented here is based on those officially reported to the Japanese regulatory authority [Pharmaceuticals and Medical Devices Agency (PMDA)] on 8 December 2009.

## METHODS

### Subjects

Because antiretrovirals are prescribed to limited number of patients in Japan and are generally administered with other antiretrovirals, a cooperative postmarketing survey [HIV-Related Drug (HRD) cooperative survey] by the pharmaceutical companies that marketed antiretrovirals has been conducted in Japan. In addition, the survey has been delegated to Nihon Ultmarc Inc. (currently CMIC-PMS Co., Ltd.). The survey has been conducted at registered sites, designed to collect safety and effectiveness information on antiretrovirals marketed in Japan. It was designed to enroll all of the patients who received antiretrovirals; however, the decisions of the enrollment were made by contractor physicians. The survey forms are filled by the contractor physicians in each fiscal year. In the present report, data from the cases registered in this survey between September 1999 and March 2008 (last follow-up was September 2009) and received Ziagen tablet are analyzed. Note that the safety data presented here is based on those officially reported to the PMDA on 8 December 2009 after the completion of mandatory postmarketing surveillance for this drug. In addition to this survey, GlaxoSmithKline K.K. conducted a detailed analysis on safety of Ziagen tablet in mothers and their children when the company was informed that Ziagen had been administered to pregnant cases during the same period in Japan.

### Observed items

The survey items were as follows: reasons for the use of Ziagen (disease names), sex, age, race, history of treatment for HIV infection, complications at the time of registration [including renal impairment (by physician's call), hepatic dysfunction (by physician's call), and hemophilia], the Centers for Disease Control and Prevention (CDC) classification,^17^ usage of Ziagen (dosage and duration of use), usage of concomitant drugs, and presence or absence of adverse events (AEs) occurring after the administration of this drug (diseases, symptoms, abnormal laboratory values, etc.), as well as onset dates, course, treatment, outcomes, and severity of AE (death, disability, hospitalization, subseriousness, congenital abnormality, etc. that are described in the Enforcement Regulation No. 253 of the Pharmaceutical Affairs Law in Japan were considered as serious AEs). The AEs were classified into five stages with regard to the causal relationship with abacavir: “definitely associated,” “associated,” “not ruled out,” “unknown,” or “ruled out” on the basis of the reports from physicians. In addition, the AEs except “ruled out” were defined as “adverse drug reactions” (ADR) in the present analysis. Moreover, both were specifically focused: (1) hypersensitivity reaction (the criteria for hypersensitivity is shown in Table 1) and (2) the usage of Ziagen in pregnant cases. Furthermore, changes in plasma virus loads (HIV-RNA copies) and CD4+ T-cell count were determined to assess the effectiveness of Ziagen.

**Table 1:** Criteria for hypersensitivity†

Category A	Hypersensitivity/anaphylactic symptoms/allergic reactions/drug allergy
Category B	Cases meeting two or more of the following items:
	• Rash
	• Fever
	• Gastrointestinal symptoms (nausea, vomiting, diarrhea, and abdominal pain)
	• Constitutional symptoms [coma, fatigue, malaise, myalgia, and abnormal chest radiographs (infiltration is mainly noted and may be localized in some cases)]
Exclusion criteria	• A patient in whom other causes are highly probable despite the presence of hypersensitivity-like symptoms
	• A patient without recurrence after readministration of abacavir
	•A patient with disappearance of symptoms during treatment with abacavir
	• A patient who does not meet the criteria for category B despite suspected hypersensitivity to abacavir

† Patients who meet the criteria for category A or B but not the exclusion criteria are determined to have hypersensitivity to abacavir.

### Statistical analysis

The *χ*^2^ test or Fisher's exact test was used for stratified analysis, and two-sided *p*-value of less than 5% was considered as significant. For multivariate analysis, logistic regression analysis was performed with stepwise selection. All of the statistical analyses were conducted using SAS Ver 9.1.3.

## RESULTS

### The number of surveyed subjects

A total of 646 subjects on Ziagen from 33 institutions participating in the HRD cooperative survey were enrolled in the present analysis; the survey forms were collected from 643 of the 646 subjects. The safety analysis included all 643 subjects. Excluding subjects who switched to ABC/3TC fixed-dose combination (Epzicom) during the observed period, 274 subjects prematurely terminated Ziagen use due to various reasons, and the resultant total observed duration was 1345.7 person-years. The effectiveness analysis included only the subjects who received Ziagen for at least 60 days and whose CD4+ T-cell count and plasma viral load (HIV-RNA copies) before and after starting Ziagen were available.

### Characteristics of the subjects

The characteristics of the subjects are shown in Table 2. Of the total 643 subjects, 593 (92.2%) were Japanese, and 581 (90.4%) were men. The age ranged from 18 to 80 years. The reason for the use of Ziagen was “HIV infection” in all the subjects, and the mean daily dose was “2 tablets (600 mg),” accounting for 640 subjects (99.5%). The number of concomitant antiretrovirals used was “2 drugs” in 335 subjects (52.1%) and “3 drugs” in 165 subjects (25.7%), implying most of whom were receiving a combination of three or more antiretrovirals. The duration of cART before the start of Ziagen was “at least 1 year and less than 2 years” in 124 subjects (22.9%) and “2 years or longer (8.4 years at the longest)” in 274 subjects (50.8%), implying most of whom were “treatment-experienced.” The mean duration of Ziagen treatment was 763.9 days, and the observed duration was 1345.7 person-years.

**Table 2:** Characteristics of the subjects

	Safety analysis population	
Patient factor		Patients (*N*)	Proportion (%)
Total		643	100.0
Reason for use	HIV infection	643	100.0
Sex	Male	581	90.4
Female	62	9.6
Age (years)	18†–64	609	94.7
65–80†	34	5.3
Ethnic groups	Japanese	593	92.2
Others	50	7.8
History of treatment with antiretrovirals	Absent	93	14.5
Present	550	85.5
History of allergy	Absent	358	55.7
Present	208	32.3
Unknown	77	12.0
Complications	Absent	91	14.2
Present	552	85.8
Renal impairment	Absent	613	95.3
Present	30	4.7
Hepatic disorder	Absent	406	63.1
Present	237	36.9
Hemophilia	Absent	541	84.1
Present	102	15.9
Concomitant use of non-anti-HIV drugs§	Absent	0	0.0
Present	643	100.0
Number of concomitant anti-HIV drugs§	None	0	0.0
1 drug	39	6.1
2 drugs	335	52.1
3 drugs	165	25.7
≥4 drugs	104	16.2
CDC classification	A	208	32.3
B	39	6.1
C	128	19.9
Unknown	268	41.7
Total duration of treatment (days)	2–180	169	26.3
181–365	76	11.8
366–730	124	19.3
731–3074	274	42.6

† Youngest age.

‡ Oldest age.

§ Total numbers throughout the observed periods.

### Adverse drug reactions

In the present analysis, as shown in Table 3, ADRs were observed in 306 (736 events) of the 643 subjects (47.6%). ADRs assessed to be “definitely associated” or “associated” with Ziagen were noted in 58 subjects with 101 incidences (9.0% of the subjects). The classes of the observed ADRs by organ were as follows: 19.0% (122/643 subjects) for “metabolism and nutrition disorders,” 18.2% (117/643) for “investigations (laboratory abnormality),” 11.8% (76/643) for “gastrointestinal disorders,” 8.4% (54/643) for “skin and subcutaneous tissue disorders,” 7.5% (48/643) for “nervous system disorders,” and 7.0% (45/643) for “hepatobiliary disorders.”

**Table 3:** Adverse drug reactions observed during the treatment with Ziagen† (736 events in 306 subjects)

Adverse drug reaction	Cases (%)
**Blood and lymphatic system disorders**	17 (2.6)
Anemia	8
Disseminated intravascular coagulation	1
Iron deficiency anemia	1
Leucopenia	1
Lymphadenopathy	1
Neutropenia	1
Pancytopenia	2
Thrombocytopenias	1
Hemorrhagic diathesis	3
**Cardiac disorders**	**6** (**0**.**9**)
Atrial fibrillation	2
Conduction disorder	1
Palpitations	3
**Endocrine disorders**	**1** (**0**.**2**)
Hypothyroidism	1
**Eye disorders**	**1** (**0**.**2**)
Conjunctival hyperemia	1
**Gastrointestinal disorders**	**76** (**11**.**8**)
Abdominal discomfort	6
Abdominal distension	1
Abdominal Pain	3
Abdominal pain upper	3
Acute abdomen	1
Ascites	1
Constipation	1
Diarrhea	21
Dyspepsia	2
Gastric ulcer	1
Gastritis	1
Gastrointestinal disorder	2
Hematochezia	1
Nausea	40
Pancreatitis acute	3
Pancreatitis relapsing	1
Stomatitis	1
Vomiting	13
Paraesthesia oral	1
**General disorders and administration site conditions**	**29** (**4**.**5**)
Asthenia	1
Chest pain	1
Fatigue	1
Feeling hot	1
Influenza like illness	1
Malaise	11
Edema	1
Pain	1
Pyrexia	11
Thirst	1
**Hepatobiliary disorders**	**45** (**7**.**0**)
Cholecystitis (acute)	1
Cholelithiasis	1
Hepatic cirrhosis	2
Hepatic failure	1
Hepatic function abnormal	26
Hepatomegaly	1
Hyperbilirubinemia	1
Jaundice	1
Liver disorder	13
**Immune system disorders**	**5** (**0**.**8**)
Drug hypersensitivity	2
Hypersensitivity	2
Immune reconstitution syndrome	1
**Infections and infestations**	**12** (**1**.**9**)
Cytomegalovirus infection	1
Hepatitis B	2
Herpes zoster	2
Nasopharyngitis	1
Pneumonia	1
Pulmonary tuberculosis	2
Sepsis	1
HIV wasting syndrome	1
Kaposi's varicelliform eruption	1
*Mycobacterium avium* complex infection	1
Anogenital warts	1
**Injury**, **poisoning**, **and procedural complications**	**1** (**0**.**2**)
Subdural hematoma	1
**Investigations**	**117**(**18**.**2**)
Alanine amino transferase increased	11
Aspartate amino transferase increased	8
Urine β-2 microglobulin increased	1
Blood amylase increased	3
Blood bilirubin increased	4
Blood cholesterol increased	4
Blood creatine phosphokinase increased	1
Blood creatinine increased	1
Blood lactate dehydrogenase increased	3
Blood lactic acid increased	6
Blood pyruvic acid increased	1
Blood triglycerides increased	34
Blood uric acid increased	25
Cardiac murmur	1
γ-glutamyltransferase increased	36
Urine glucose present	1
Granulocyte count decreased	1
Hemoglobin decreased	2
Liver function test abnormal	4
Lymphocyte count increased	2
Neutrophil count decreased	1
Platelet count decreased	8
Red blood cell count decreased	1
Red blood cell count increased	1
Weight decreased	1
White blood cell count decreased	10
Blood alkaline phosphatase increased	10
Hepatic enzyme increased	1
**Metabolism and nutrition disorders**	**122** (**19**.**0**%)
Anorexia	3
Diabetes mellitus	11
Hypercholesterolemia	5
Hyperglycemia	3
Hyperlactacidemia	4
Hypertriglyceridemia	17
Hyperuricemia	5
Hypophosphatemia	1
Lactic acidosis	1
Fat redistribution	1
Decreased appetite	1
Hyperlipidemia	78
Hyperamylasemia	1
**Musculoskeletal and connective tissue disorders**	**11** (**1**.**7**)
Arthralgia	2
Muscular weakness	1
Myalgia	3
Osteonecrosis	2
Osteoporosis	1
Rhabdomyolysis	1
Musculoskeletal stiffness	1
**Neoplasms benign**, **malignant**, **and unspecified** (**including cysts and polyps**)	**1** (**0**.**2**)
Kaposi's sarcoma	1
**Nervous system disorders**	**48** (**7**.**5**)
Burning sensation	1
Cerebral hemorrhage	2
Cerebral infarction	1
Convulsion	3
Disturbance in attention	1
Dizziness	11
Dysgeusia	2
Guillain–Barre syndrome	1
Headache	9
Hyperesthesia	1
Hypoesthesia	13
Nervous system disorder	1
Neuropathy peripheral	9
Somnolence	4
Tremor	1
Facial nerve disorder	1
**Psychiatric disorders**	**21** (**3**.**3**)
Abnormal dreams	5
Depression	4
Emotional disorder	1
Insomnia	11
Mental disorder	3
Terminal insomnia	1
**Renal and urinary disorders**	**8** (**1**.**2**)
Nephrotic syndrome	1
Neurogenic bladder	2
Renal disorder	2
Renal failure acute	1
Renal failure chronic	1
Renal impairment	5
**Reproductive system and breast disorders**	**5** (**0**.**8**)
Gynecomastia	5
**Respiratory**, **thoracic**, **and mediastinal disorders**	**13** (**2**.**0**)
Asthma	2
Cough	1
Dyspnea	4
Hyperventilation	2
Interstitial lung disease	2
Pulmonary embolism	1
Upper respiratory tract inflammation	1
**Skin and subcutaneous tissue disorders**	**54** (**8**.**4**)
Drug eruption	7
Eczema	1
Erythema	1
Erythema multiforme	1
Lipoatrophy	5
Pain of skin	1
Rash	26
Rash generalized	1
Seborrheic dermatitis	1
Lipodystrophy acquired	7
Partial lipodystrophy	1
Facial wasting	2
Hypoesthesia facial	1
**Vascular disorders**	**11** (**1**.**7**)
Hypertension	10
Thromboangiitis obliterans	1
Deep vein thrombosis	1
Hemorrhage	1

† The terms are based on Medical Dictionary for Regulatory Activities version 12.0.

The names of ADRs were converted to the preferred terms by referring the Medical Dictionary for Regulatory Activities version 12.0, on the basis of the descriptions provided by the physicians. Similar events (e.g., abnormal hepatic function and liver disorders, and rash and drug eruption) were separately compiled. Consequently, the major individual ADRs were as follows: 78 incidences of “hyperlipidemia,” 40 incidences of “nausea,” 36 incidences of “increased γ-glutamyltransferase,” 34 incidences of “increased blood triglycerides,” 26 incidences of “abnormal hepatic function,” 26 incidences of “rash,” and 25 incidences of “increased blood uric acid.” Serious ADRs were reported in 65 subjects with 104 incidences, including “abnormal hepatic function” and “decreased white blood cell count” in five cases each; “rash” in four cases; “convulsion,” “renal impairment,” “diabetes mellitus,” “fever,” and “decreased platelet count” in three cases each; and “hepatic cirrhosis,” “liver disorder,” “osteonecrosis,” “nausea,” “diarrhea,” “acute pancreatitis,” “interstitial lung disease,” “cerebral hemorrhage,” “hyperlipidemia,” and “drug eruption” in two cases each. Among these serious ADRs, 15 events in 12 subjects were assessed to be “definitely associated” or “associated” with Ziagen administration, whereas association with Ziagen in any of the other incidences was classified as “not ruled out” or “unknown.” Although the association between abacavir and myocardial infarction (MI) has been reported,^18^ such association has been denied by other studies, as well as by a recently published meta-analysis performed by the Food and Drug Administration of the USA.^19^–^21^ Among the patients involved in the present analysis, there was no report of ischemic heart diseases, including MI and angina pectoris. The outcomes of the cases in which these serious ADR occurred were death in three subjects (“ascites and hepatic cirrhosis,” “decreased platelet count,” and “interstitial lung disease” in one subject each) and sequelae in two subjects (“cerebral infarction” and “osteonecrosis” in one subject each; Table 4). Although all the cases of death and sequelae were suspected to be associated with the primary diseases or pre-existing complications, their association with Ziagen was classified as “unknown” or “not ruled out.”

**Table 4:** Cases of death/sequelae

Sex/age	Previous complications	Adverse Drug Reactions	Treatment duration	Outcome	Association with Ziagen	Items assessed as “associated”
Man in his 40s	Hemophilia A, chronic hepatitis C, and diabetes mellitus	Ascites Hepatic cirrhosis	6 months	Death	“Not ruled out”	Hepatitis C
Man in his 20s	Hemophilia A, chronic hepatitis C, and femoral head necrosis	Osteonecrosis†	Approximately 3 years	Sequela	“Not ruled out”	AIDS
Platelet count decreased/Hemorrhage Intracranial	Approximately 5 years 6 months	Death	“Not ruled out”	AIDS/hemophilia
Man in his 60s	none	Interstitial lung disease	Occurred 3 months after discontinuation	Death	“Unknown”	
Man in his 60s	Hypertension, Sequela of cerebral infarction	Cerebral infarction†	3 years	Sequela	“Unknown”	

† Deterioration of previously existing femoral head necrosis.

‡ Another episode of cerebral infarction.

### Adverse drug reactions stratified by characteristics of the subjects

The effects of following background factors on the ADR were analyzed: sex, pregnancy, age, race, history of HIV treatment, duration of HIV infection, history of allergy, renal impairment (at the baseline), hepatic dysfunction (at the baseline), hemophilia, other complications, the number of concomitant antiretrovirals, CDC classification, treatment for opportunistic infection, total duration of Ziagen administration, and total dosage of Ziagen. In addition, statistically significant differences were observed with regard to “presence or absence of history of allergy,” “the number of concomitant antiretrovirals,” “hemophilia,” and “CDC classification” with univariate analysis (Table 5); all of which except “CDC classification” were found to be associated with ADR with multivariate analysis (Table 6).

**Table 5:** The frequency of adverse drug reactions (ADRs) according to the characteristics of the subjects

Factors		No. of patients	with ADR	No. of ADR events	Incidence of ADR (%)	*χ*‡ or Fisher's exact test (based on cases)
Overall		643	306	736	47.6	–
Sex	Male	581	276	672	47.5	NS¶
Female	62	30	64	48.9
Age	Adult†	609	289	698	47.5	NS
Elderly‡	34	17	38	50.0
Ethinic groups	Japanese	593	288	703	48.6	NS
Others	50	18	33	36.0
History of treatment with antiretrovirals	Absent	93	49	115	52.7	NS
Present	550	257	621	46.7
History of allergy	Absent	358	152	355	42.5	*p* = 0.004*
Present	208	115	307	55.3
Unknown	77	39	74	50.6	
Complications	Absent	91	40	74	44.0	NS
Present	552	266	662	48.2
Renal impairment	Absent	613	293	697	47.8	NS
Present	30	13	39	43.3
Hepatic disorder	Absent	406	187	380	46.1	NS
Present	237	119	356	50.2
Hemophilia	Absent	541	244	532	45.1	*p* = 0.005*
Present	102	62	204	60.8
Number of concomitant anti-HIV drugs§	1 drug	39	11	24	28.2	*p* = 0.007*
2 drugs	335	155	333	46.3
3 drugs	165	78	174	47.3
4 drugs ≤	104	62	205	59.6
CDC classification	A	208	94	224	45.2	*p* = 0.003*
B	39	29	75	74.4
C	128	61	164	47.7
Unknown	268	122	273	45.5	

† 18 to 64 years.

‡ Elderly patients.

§ Total numbers throughout the observed periods.

¶ NS, no statistical significance.

* *p* < 0.05.

**Table 6:** The background characteristics that are associated with the frequency of ADR†

Factors	Odds ratio	95% confidence interval
History of allergy	1.65	1.18–2.31
Hemophilia	1.84	1.18–2.86
Number of concomitant anti-HIV drugs	1.21	1.05–1.40

† Only factors that have shown association with the frequency of ADR are listed. For History of Allergy, “unknown” was regarded as “absent”; for CDC classification, “unknown” was regarded as “category A.”

The incidence of ADR in patients with a history of allergy was 55.3% (115/208 subjects) and significantly higher than that in those without a history of allergy (42.5% [152/358]; *p* < 0.01). It seemed to have been mainly driven by “rash”(*p* = 0.004). The serious ADR were reported in several patients with a history of allergy (“rash” in four cases, “decreased white blood cell count” in three cases, and “abnormal hepatic function” in two cases).

The incidence of ADR in hemophilia patients was 60.8% (62/102 subjects) and significantly higher than that in non-hemophilia patients [45.1% (244/541); *p* < 0.01]. However, no apparent trend was observed in the types of ADR. Serious ADRs were reported in 27 hemophilia subjects with 49 incidences. The most common ADR were as follows: “abnormal hepatic function,” “renal impairment,” and “decreased white blood cell count” in three patients each; and “rash,” “decreased platelet count,” “fever,” “acute pancreatitis,” “hepatic cirrhosis,” and “liver disorder” in two patients each.

The incidence of ADR was significantly higher in subjects with more concomitant use of antiretrovirals [28.2% (11/39) in subjects receiving “1 drug,” 46.3% (155/335) in those receiving “2 drugs,” 47.3% (78/165) in those receiving “3 drugs,” and 59.6% (62/104) in those receiving “4 or more drugs”], indicating the effects of the combined use of multiple antiretrovirals.

The incidence of ADRs according to CDC Classification of HIV Infection at the baseline was 45.2% (94/208) for “category A,” 74.4% (29/39) for “category B,” and 47.7% (61/128) for “category C,” showing significant differences (*p* < 0.01). Although the incidence was higher in those with category B, no apparent difference was observed between the classes of ADR. However, this association was not found with multivariate analysis perhaps because of a strong relation between CDC classification and the number of concomitant anti-HIV drugs (Chi-square test, *p* = 0.0035).

### Hypersensitivity

The onset of hypersensitivity was analyzed according to the criteria shown in Table 1, on the basis of the survey forms filled by physicians. This analysis suggested that hypersensitivity reaction occurred in 15 patients (Table 7), and the incidence rate was 2.3% (15/643). Although human leukocyte antigen (HLA) B*5701 has been revealed to be strongly associated with hypersensitivity to abacavir,^22^,^23^ this allele is rarely expressed in Japanese individuals.^24^–^27^ However, all subjects reported were Japanese. Note that the diagnostic criteria for hypersensitivity to abacavir in clinical practice is in accordance with criteria B in Table 1,^28^ and “allergic reaction” and “drug allergy” included in criteria A encompass a wide range of pathological conditions; thus, the present survey might have included a considerable number of cases that might not be true hypersensitivity. When the individual cases were examined (Table 7), the cases reported to be “associated” or “definitely associated” with Ziagen administration accounted for 1.7% (11/643). The suspected drugs used concomitantly were reported in 7 of 15 patients: two of the seven patients had nevirapine that is known to be associated with hypersensitivity; the other two patients received efavirenz, which is known to frequently cause rash. All the four subjects with “serious” hypersensitivity were reported to be “recovery” or “amelioration.”

**Table 7:** Patients presented hypersensitivity

	Adverse reaction	Association	Severity	Outcome	time to onset (days)	Sex	Age (year)	Complications	Concomitant suspected products †	Comment from physicians
1	Hypersensitivity	Associated	Not serious	Ameliorated	6	Male	30			
2	Erythema multiforme	Associated	Serious	Ameliorated	8	Female	32	Hepatitis C Gastric ulcer	ddI, d4T	Association with ddI and d4T cannot be ruled out
3	Rash/Pyrexia	Not ruled out/Definitely associated	Not serious	Ameliorated	9	Male	37	Hemophilia A Hepatitis C	NVP	
4	Drug eruption/Myalgia	Definitely associated/Not ruled out	Not serious	Recovered	11/13	Female	63		d4T, EFV, 3TC	
5	Rash, Pyrexia	Not ruled out	Serious	Recovered	57	Male	31	Hepatitis C Hemophilia A	NVP	Association with NVP cannot be ruled out
6	Upper respiratory tract inflammation	Not ruled out	Not serious	Recovered	13	Male	23	Hemophilia B Hepatitis C		
7	Rash, Headache/Anorexia	Associated/Not ruled out	Not serious	Ameliorated	2	Male	36	Hepatitis B Syphilis	AZT/3TC	
8	Drug hypersensitivity	Associated	Not serious	Recovered	20	Male	44			
9	Rash	Not ruled out	Not serious	Ameliorated	10	Male	38		EFV,LPV/rtv	Association with EFV and LPV/rtv could not be ruled out
10	Pyrexia, vomiting and diarrhea	Not ruled out	Not serious	Ameliorated	12	Male	34		AZT/3TC	
11	Pyrexia, Myalgia, Abdominal pain	Associated	Serious	Recovered	2	Male	39	Hepatitis C Hemophilia B		The symptoms were improved after changing ABC to ddI
12	Queasy, Pyrexia, Myalgia	Associated	Not serious	Recovered	1	Male	35	Hepatitis B Syphilis		The symptoms disappeared after discontinuation of ABC
13	Drug hypersensitivity	Associated	Not serious	Recovered	16	Male	62	Hypertension		
14	Rash, Conjunctival hyperemia, Pyrexia	Definitely associated	Not serious	Ameliorated	11	Male	31			
15	Drug eruption	Associated	Serious	Ameliorated	12	Female	60s	Hypertension		

† ddI, didanosine; d4T, stavudine; AZT, zidovudine; NVP, nevirapine; 3TC, lamivudine; LPV/rtv, lopinavir/ritonavir; TDF, tenofovir disoproxil fumarate; EFV, efavirenz.

### Duration of Ziagen treatment prior to onset of adverse drug reactions

The duration from initiation of Ziagen treatment to onset of ADR were known in 617 of 736 events in 306 patients. Of these, 230 events (37.3%) were occurred within 28 days after the start of Ziagen administration; 434 events (70.3%) occurred within 365 days (1 year); 183 events (29.7%) occurred after the 366 days. The frequent ADR within 28 days included “nausea,” “rash,” “hyperlipidemia,” “vomiting,” and “dizziness”; and the frequent ADR occurred within 365 days after Ziagen administration included “hyperlipidemia,” “increased blood triglycerides,” “increased γ-glutamyltransferase,” and “increased blood uric acid.”

### Pregnant cases

During the survey period, information on three pregnant Japanese patients receiving Ziagen tablet and two of their offspring was obtained. One of them had been on Ziagen before the pregnancy, and the other two started Ziagen in their second trimesters. Of the three patients, one developed mild “hyperlipidemia” during pregnancy and at the time of delivery, and their associations with Ziagen were not ruled out. The consequences of the pregnancy were two deliveries and one induced abortion (one of the two who had started Ziagen therapy from the second trimesters). Both of the deliveries were cesarean sections, and both the neonates were normal. The reason or cause for the induced abortion was unknown. In one of the two neonates who could be followed up, “infantile apneic attack” on the day of birth, “fever” and “rash” on the fourth day after birth (HLA class I type was unknown), and “anemia” approximately 1 month after birth were reported with “unknown” association with Ziagen to the mother; all events were mild and improved.

### Effectiveness analysis

**Figure 1:**
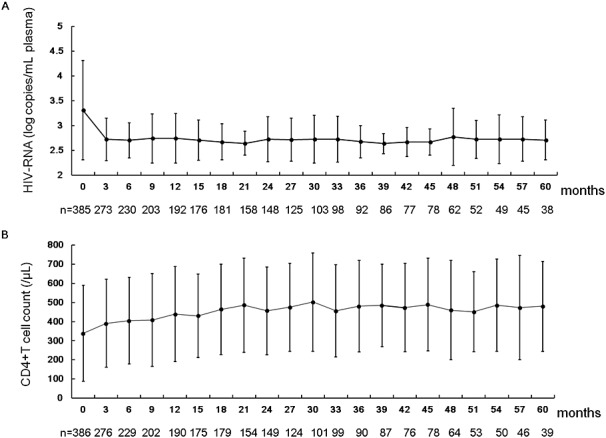
Change in plasma viral load and peripheral CD4+ T-cell count after the initiation of Ziagen therapy

The effectiveness analysis was performed on the subjects who received Ziagen tablet for at least 60 days and whose plasma viral load and CD4+ T-cell count before and after initiation of Ziagen treatment were available. The maximal duration of Ziagen administration was 3074 days (8.4 years). Note that, as shown in Table 2, 85.5% of HIV-positive subjects included in the present analysis had a history of antiretroviral treatment before starting Ziagen. The mean plasma viral load (HIV-RNA copies) was decreased from 3.3 ± 1.0 log copies/mL (mean ± standard deviation [SD]) before treatment to 2.7 ± 0.4 log copies/mL 3 months after the start of Ziagen administration (Figure 1A). Note that although the current detection limit of HIV-1 RNA quantification is 20–50 RNA copies/mL, it used to be 400 RNA copies/mL during the early phase of this survey; therefore, all of the values under 400 were uniformly treated as 399. The mean CD4+ T-cell count was increased from 339.1 ± 251.0/μL before treatment to 508.8 ± 264.9/μL 30 months after the start of Ziagen (Figure 1B).

## DISCUSSION

In the present analysis, the safety and effectiveness of ABC (Ziagen 300 mg) in Japanese HIV+ patients were assessed. To our knowledge, this is the largest and longest survey for monitoring the ADRs associated with abacavir in East Asians. Because abacavir is recommended as a part of the preferred nucleoside reverse transcriptase inhibitor backbone of initial anti-HIV therapy in the guideline of the Ministry of Health, Labor, and Welfare in Japan (2012 version)^16^ and in the guideline issued by the IAS-USA,^13^ the results of the present survey would contribute to the understanding of safety of abacavir in the Japanese and East Asian populations.

Although serious ADRs were observed in 65 subjects with 104 incidences, only ADR observed in 12 subjects (1.87%) were assessed to be “definitely associated” or “associated” with abacavir. In addition, abacavir seemed not to have been associated with any of the three cases of fatal outcome. These results suggest that abacavir can be safely used in Japanese HIV+ people.

Although associations between abacavir and acute MI^18^ have been denied by recent meta-analyses,^29^ it has been one of the reasons why the regimens containing abacavir are categorized as “alternative” in the DHHS guideline.^14^ The fact that there was no report of ischemic heart diseases, will inform to healthcare professionals regarding the safety of abacavir in East Asian populations.

Because the frequency of HLA-B*5701 allele is extremely low (0–0.1%) in the Japanese population,^24^–^27^ the risk for hypersensitivity to abacavir in Japanese has been considered low. It was found that 15 subjects (2.3%) had met the criteria for the survey-defined “hypersensitivity reaction.” However, this frequency was substantially lower than that reported in the White population (9–10%)^22^,^30^ and that (4%) reported in B*5701 negative White individuals.^22^ Perhaps, not all of the 15 subjects had true hypersensitivity to abacavir. According to a recent report from South Korea, where the frequency of HLA-B*5701 is almost 0% as well, although “clinical” hypersensitivity was observed in 5% (7/150) of the subjects, none of them could be confirmed by skin patch test, suggesting that “true” hypersensitivity was rare among Korean patients.^31^ Similarly, because the present survey aimed to widely collect safety information, false cases, such as simple “allergic reaction” or “drug allergy,” might have been included. In addition, some patients received concomitant suspected products such as nevirapine and efavirenz that might have caused hypersensitivity or rash. Therefore, although the frequency of true hypersensitivity to abacavir could not be accurately determined, we believe the frequency is substantially low; however, careful follow-up is needed for patients started abacavir within the first several weeks.

During the follow-up period, three pregnant cases received ABC, but the number was too small to conclude safety of ABC in pregnancy. Although the Antiretroviral Pregnancy Registry reports no apparent increase in birth defects thus far (http://www.apregistry.com/forms/exec-summary.pdf), careful monitoring is warranted when ABC is used in pregnant women.

In conclusion, we conducted a postmarketing surveillance for Ziagen tablet to collect information on the safety and effectiveness of this drug in clinical practice. There were no new issues that needed to be investigated with regard to the safety. We are planning to collect further information on the safety and effectiveness of abacavir in Japanese population, by analyzing data from postmarketing surveillance for Epzicom, an abacavir–lamivudine fixed-dose combination.

## CONFLICT OF INTEREST

Tomoko Kurita, Tomomi Kitaichi, Takako Nagao, Toshiyuki Miura, and Yoshifumi Kitazono are employees of ViiV Healthcare K. K.

## KEY POINTS

- Abacavir sulfate tablet can be relatively safely taken by Japanese HIV+ people.
- Hypersensitivity reaction to abacavir is considerably low in Japan.
- Incidence of myocardial infarction associated with abacavir seemed low in Japan.

## ETHICS STATEMENT

This was a surveillance mandated by the Japanese regulatory authority to collect information from all of the patients receiving the drug. IRB approval were obtained where appropriate according to institutional rule.
